# Use of Antiandrogens as Therapeutic Agents in COVID-19 Patients

**DOI:** 10.3390/v14122728

**Published:** 2022-12-07

**Authors:** Efstathios S. Giotis, Emine Cil, Greg N. Brooke

**Affiliations:** 1 Department of Infectious Diseases, Imperial College London, London W2 1PG, UK; 2 School of Life Sciences, University of Essex, Colchester CO4 3SQ, UK

**Keywords:** coronavirus, TMPRSS2, ACE2, SARS-CoV-2, androgens, sex hormones, antiandrogens

## Abstract

COVID-19, caused by the severe acute respiratory syndrome coronavirus 2 (SARS CoV-2), is estimated to have caused over 6.5 million deaths worldwide. The emergence of fast-evolving SARS-CoV-2 variants of concern alongside increased transmissibility and/or virulence, as well as immune and vaccine escape capabilities, highlight the urgent need for more effective antivirals to combat the disease in the long run along with regularly updated vaccine boosters. One of the early risk factors identified during the COVID-19 pandemic was that men are more likely to become infected by the virus, more likely to develop severe disease and exhibit a higher likelihood of hospitalisation and mortality rates compared to women. An association exists between SARS-CoV-2 infectiveness and disease severity with sex steroid hormones and, in particular, androgens. Several studies underlined the importance of the androgen-mediated regulation of the host protease TMPRSS2 and the cell entry protein ACE2, as well as the key role of these factors in the entry of the virus into target cells. In this context, modulating androgen signalling is a promising strategy to block viral infection, and antiandrogens could be used as a preventative measure at the pre- or early hospitalisation stage of COVID-19 disease. Different antiandrogens, including commercial drugs used to treat metastatic castration-sensitive prostate cancer and other conditions, have been tested as antivirals with varying success. In this review, we summarise the most recent updates concerning the use of antiandrogens as prophylactic and therapeutic options for COVID-19.

## 1. Emergence of COVID-19 and Variants of Concern

Coronaviruses (CoV) are a large family of positive-sense, enveloped, single-stranded RNA viruses that are common in people and many animal species. Human coronaviruses (hCoV) include the common cold CoV 229E, OC43, NL63 and HKU1, which predominantly cause mild respiratory illness, and three highly pathogenic coronaviruses, which cause more severe and fatal diseases in people, e.g., severe acute respiratory syndrome coronavirus (SARS-CoV, 2003), Middle East respiratory syndrome coronavirus (MERS-CoV, 2012) and more recently, SARS-CoV-2 (2019), the causative agent of COVID-19 [1]. At the end of September 2022, over 600 million cases of COVID-19 were confirmed globally, with a 4–5% crude mortality risk (deaths per 100 patients hospitalized primarily for COVID-19) [2]. COVID-19 may present with mild, moderate or severe symptoms in patients. Mild symptoms include fever, dry cough, dyspnoea and fatigue, while COVID-19-associated mortality is attributed to severe respiratory failure resembling acute respiratory distress syndrome (ARDS) [3,4,5]. Severe pneumonia COVID-19 patients exhibit exacerbated inflammatory response resulting in excessive release of pro-inflammatory cytokines, known as the “cytokine storm”, which leads to alveolar damage, fibrosis and progressive respiratory failure [5,6,7,8]. It is becoming increasingly clear that COVID-19 is a multisystem disease and can also lead to heart failure, acute kidney failure and neurovascular injuries [5,9,10]. In addition, growing patient testimony and scientific evidence demonstrate that a large number of patients (approximately 2% of the UK population alone) also suffer from post-COVID syndrome (long COVID or post-acute sequelae of COVID-19 (PASC)), which causes debilitating physical or mental symptoms for long periods after infection [5,11,12,13,14].

Vaccination programmes have been key to reducing COVID-19 hospital admissions and mortality rates, but for some countries and groups of people, the uptake is still low due to various socioeconomic factors and accessibility inequalities. In addition, *vaccination*-acquired *immunity wanes* substantially with time and boosters are required to restore the efficiency of vaccines against severe diseases [15,16]. Despite progress in countries with high vaccination rates, the pandemic is still actively unfolding with emerging virus variants that can evade vaccine or natural immunity triggering new public health challenges. Virus variants are characterised by the WHO as variants of concern (VOC) if they demonstrate increased transmissibility, virulence or cause reduced effectiveness of vaccine-induced protection, diagnostic tests and management measures [17,18]. Important variants include B.1.1.7 (Alpha), B.1.351 (Beta), P.1 (Gamma) and B.1.617.2 (Delta) [18,19,20]. Two new sub-variants (B.1.1.529; Omicron) designated as BA.4 and BA.5 emerged in December 2021 and January 2022, respectively, which replaced the other circulating Omicron lineages and have driven the current wave of infections in Africa, the United States and Europe [21]. These two subvariants appear more contagious and circumvent immunity from previous infections or vaccination [22,23]. As a result of the emergence of Omicron variants, several updated COVID-19 vaccines have been developed and approved for use in the United States, United Kingdom and elsewhere. However, a recent study suggested that the direct protective benefit of the Omicron-updated bivalent vaccines is only marginal compared to ancestral-based vaccines [24]. 

COVID-19 hospitalised patient management in high-income countries is largely focused on highly efficient albeit high-priced monoclonal antibodies [25], anti-inflammatory drugs such as dexamethasone, which reduce cytokine levels and immune-mediated pathogenesis in the hyper-inflammatory phase of the disease [26], and/or drugs which directly interfere with viral replication. The latter act either by inhibition of the viral 3CL^pro^ protease (paxlovid), by inhibition of the RNA-dependent RNA polymerase (remdesivir) [27] or by introducing copying errors in the viral genome (molnupiravir) [28]. Recent clinical studies reported that remdesivir has no significant effect on patients who are already being ventilated [29] and that some paxlovid- or molnupiravir-treated patients experience rebound infections after completing a course of treatment [30,31]. Taken together, more efficient and less expensive antiviral drugs for pre-emptive protection against emerging variants, reducing reliance on boosters, are required, especially in low- and middle-income countries.

## 2. Risk Factors and Gender Disparity in COVID-19 Patients

Most COVID-19 patients are expected to have a favourable prognosis. However, some groups are at higher risk of developing severe or fatal illness due to underlying health conditions. These include demographic factors (i.e., older age), gender, occupational exposure, ethnicity and, more importantly, comorbidities such as diabetes, hypertension, obesity, neurological disorders, cancer, heart, lung, liver and kidney disease [32,33,34,35,36]. Researchers are in the early stages of understanding the risk factors of viral persistence and long COVID. Accumulating evidence shows that people who experienced multisystem inflammatory syndrome (MIS) with underlying health conditions and who were not vaccinated were more likely to develop long-term post-COVID sequelae [37,38,39].

Since the *start* of the coronavirus *pandemic*, emerging gender-disaggregated data from multiple countries suggested that men were disproportionally affected by COVID-19. Initially, it was hypothesised that the higher occupational exposure and comorbidities of males compared to females were contributory to this discrepancy. Follow-up studies confirmed that men globally exhibit higher infection, hospitalisation and mortality rates compared to women [40,41,42]. Female COVID-19 patients were also found to clear the virus and recover substantially earlier compared to male patients [43,44]. Sex differences in prevalence, severity and outcome of viral infection were previously reported for other viruses such as influenza viruses, HIV, herpes simplex viruses, hepatitis B, measles and West Nile virus [45,46,47]. The reasons accounting for the higher vulnerability of males to viruses are not clear, but several immunological, as well as genetic, hormonal and socio-behavioural explanations have been put forward. 

The innate immune system serves as the first line of host defence against invading viruses and involves sensing viruses through pattern recognition receptors and activating inflammatory pathways, which eventually result in viral clearance. Innate immune cells, i.e., monocytes, dendritic cells and macrophages, are functionally more active in females [48,49,50]. Females are typically able to launch more vigorous innate and adaptive immune responses, have higher resistance to most viral infections and exhibit higher antibody responses following infection and vaccination compared to males [48,51,52,53]. The bi-allelic expression of immune-related genes present on the X chromosome, such as the RNA receptors TLR7 and TLR8, may be responsible for sexually dimorphic immunophenotypes [52,53,54]. Hormonal regulation may also be responsible for the gender disparity in disease progression and outcomes following viral challenges. Steroid hormones, such as androgens, oestrogens, progesterone and glucocorticoids, control the activity of innate immune cells and the survival and differentiation of T and B lymphocytes in both men and women [48,54,55,56]. Many studies have been published on the effects of steroid hormones on immune responses. The consensus is that androgens (primarily male hormones) are predominantly immunosuppressive while oestrogens (the primary female hormones) are mostly immunoprotective [49,50,57,58,59,60], and they can thus potentially suppress or enhance antiviral responses, respectively. In addition to their effects on antiviral responses, androgens can also directly influence virus activity, i.e., HBV and HCV [61]. Conversely, viruses are also able to manipulate sex steroid receptor signalling mechanisms to serve their own survival and enhance their replication rate [62]. The extent to which male and female immune responses differ during SARS-CoV-2 infection and the possibility of a bi-directional interaction between the virus and androgens have been overlooked so far and deserve greater attention.

## 3. Cell Entry of SARS-CoV-2

**Figure 1:**
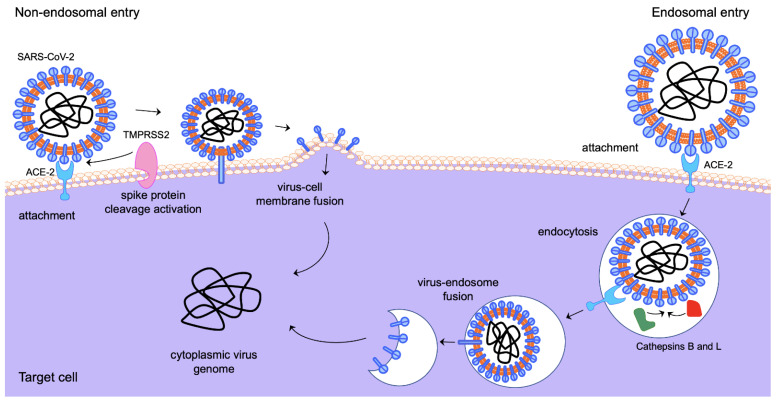
Alternative entry routes of SARS-CoV-2 into target cells. Elements of the schematic are from Motifolio.

Viral attachment, fusion and entry of SARS-CoV-2 into target cells are mediated by the virus’ envelope spike (S) glycoprotein [63,64,65]. The S protein is the *main viral antigenic* site for inducing antibody responses and is, therefore, a key target for the development of antivirals and vaccines. S is synthesised as a precursor protein that is cleaved into two subunits: the N-terminal S1 subunit, which contains the receptor binding domain, and the C-terminal S2 subunit, which harbours the viral fusion machinery [63,64,66]. SARS-CoV-2 initiates cell entry by binding its S to the transmembrane glycoprotein Angiotensin-Converting Enzyme 2 (ACE2), a negative controller of the Renin–Angiotensin system [67,68]. Following binding, the virus gains access to the cell by two alternative routes (Figure 1), either via direct fusion with the cell membrane or via clathrin-mediated endocytosis [64,65,66]. Both entry mechanisms require priming of the S protein by proteolytic cleavage at two sites in a manner reminiscent of that of the proteolytic cleavage and maturation of influenza A viruses’ entry protein haemagglutinin [69,70]. The first cleavage site is a polybasic insertion (PRRAR) at the S1–S2 boundary and the second cleavage occurs at the S2′ site in the S2 subunit, a crucial step for triggering the fusion of viral and host cell membranes [64,65,71,72]. The S1–S2 site is cleaved by the serine *protease* furin, which is ubiquitously expressed in the respiratory tract, while the S2′ site can be cleaved by either the endosomal cathepsins (B and L) in the endolysosome or by the cell-surface transmembrane protease serine 2 (TMPRSS2) at the plasma membrane [63,73,74]. TMPRSS2 is part of the type 2 transmembrane serine protease (TTSP) family and has been extensively studied in the context of prostate cancer as its expression is regulated in response to androgens through direct transcriptional regulation by the androgen receptor (AR) [75,76]. Other TTSPs, such as TMPRSS4, may also act as SARS-CoV-2 cell entry mediators [77]. The aftermath of SARS-CoV-2 cell entry is governed by a complicated network of host–pathogen interactions, where a range of viral and host factors can demarcate the disease outcome. 

## 4. ACE2 and TMPRSS2 as Candidate Targets for Antiviral Therapy

A large number of viral and host factors that play key roles in virus pathogenesis have been discovered. Among them, ACE2 and TMPRSS2 have been proposed as the most attractive targets for the development of antivirals that could inhibit or delay virus infection, thus possibly reducing viral transmission and symptom severity. Both factors are expressed in the nasal and bronchial secretory cells, including alveolar epithelial type II cells (AT2), which is the main target cell subpopulation of SARS-CoV-2, as well as gastrointestinal and other virus-targeted tissues [78,79,80,81,82]. ACE2 regulates key processes in the human body, such as blood pressure, cardiovascular function, wound healing and inflammation and acts as the main cellular receptor for SARS-CoV and hCoV NL63 [83,84]. TMPRSS2 is also essential for the cell entry of other viruses, i.e., influenza A and B, HCV, as well as other coronaviruses [85,86,87,88,89]. 

At the onset of the COVID-19 pandemic, there were studies suggesting that higher ACE2 and TMPRSS2 levels may be associated with higher susceptibility to SARS-CoV-2 infection, and this spurred extensive research that unveiled novel insights into the function and biology of ACE2/TMPRSS2. Several reports revealed an association between the variable expression of ACE2 and TMPRSS2 in different tissues across individuals with COVID-19 severity/fatality variations [90,91,92,93,94]. The lung airway expression of both ACE2 and TMPRSS2 is lower in children who are less susceptible to infection compared with adults, and significantly higher in smokers compared to non-smokers and in patients with chronic obstructive pulmonary disease (COPD) compared to healthy individuals [95,96]. 

Targeting ACE2 was considered the obvious first-choice target for prophylactic and therapeutic interventions aiming to block virus accessibility to respiratory cells early on in infection [97,98]. Several strategies have been evaluated in vitro and in vivo with varied success, including decoy or soluble ACE2 molecules, pseudoligands with a high ACE2 affinity and blocking antibodies [67,99,100]. However, a disadvantage of these approaches is the potential dysregulation of ACE2-mediated vasodilation, amino acid transport and pancreatic insulin secretion [100,101,102,103]. *TMPRSS2-inhibitors* are arguably better candidates for COVID-19 antivirals than those for ACE2 as knockout of TMPRSS2 protein causes no overt detrimental phenotype [104,105]. Numerous TMPRSS2 inhibitors have been tested in in vitro studies that showed they inhibit entry of SARS-CoV-2 in lung cells, including notably camostat, nafamostat and several peptidomimetic inhibitors [67,106,107,108,109]. These inhibitors were either ‘repurposed’ commercially available drugs, designed de novo or identified by virtual screenings and have been reviewed extensively elsewhere [110,111,112,113,114,115]. Several of these compounds have already been or are currently being investigated in randomised, controlled trials to assess their use as monotherapies against COVID-19. Clinical trials testing the efficacy of camostat and nafamostat have shown contradictory results. Some studies demonstrated a clear, beneficial effect for COVID-19 patients [116,117,118], while others showed only modest clinical improvements [107]. A possible caveat for these inhibitors is that the currently prevailing Omicron subvariants favour cell entry via the endosomal pathway [119,120,121], and it is possible that inhibitors targeting the TMPRSS2 (non-endosomal) entry route might not have a significant effect. Separate studies have targeted the expression of TMPRSS2 using either androgen receptor (AR) antagonists or drugs that lower circulating androgen levels. The relationship between androgens and COVID-19 is not entirely clear and is even considered controversial by some researchers. Conflicting studies have been published reporting that both subdued or excessive testosterone levels can lead to severe COVID-19 disease, e.g., in hypogonadism in older men and testosterone-treated postmenopausal women, respectively, which suggests that the androgen-mediated effects of COVID-19 patients are likely multidimensional and interdependent on a range of confounding factors [122].

## 5. Androgen Receptor Structure and Signalling

The androgen receptor (AR), a member of the steroid receptor family, is a ligand-dependent transcription factor with a modular structure [123]. The N-terminal activation function (AF-1) is a region that mediates protein–protein interactions and is important for transcriptional activity. Adjacent to this region is the DNA binding domain (DBD), consisting of two zinc-finger-like modules, which modulate the interaction of the receptors with specific DNA response elements present in the regulatory regions of target genes. The C-terminus houses the ligand binding domain (LBD)/activation function 2 (AF2). The AR LBD is formed of 12 α-helices which fold to form a pocket into which androgen fits. Ligand binding promotes a conformational change, resulting in receptor activation [124,125]. 

**Figure 2:**
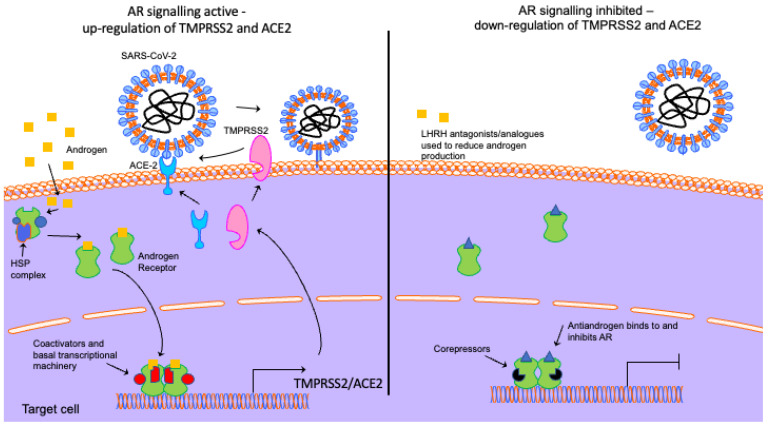
Androgen receptor signalling and how androgen deprivation therapies might block viral uptake. In the absence of androgen, the AR is located in the cytoplasm bound to a heat shock protein (HSP) complex. Ligand binding promotes dissociation of this complex, nuclear localisation and dimerisation. The AR binds to androgen response elements in the regulatory regions of target genes (e.g., TMPRSS2 and ACE2) and, through the recruitment of coactivators and the basal transcriptional machinery, regulates transcription. LHRH analogues/antagonists reduce androgen production to inhibit AR signalling. Antiandrogens bind to and inhibit the AR. The downregulation of AR signalling reduces TMPRSS2 and ACE2 expression, reducing COVID-19 entry. Elements of the schematic are from Motifolio.

In the absence of a ligand, the AR is located in the cytoplasm and is held in a ligand-binding competent state by a heat shock protein complex (Figure 2). Testosterone can diffuse into the cell and is converted to the more potent androgen dihydrotestosterone (DHT) by 5α-reductase. Upon ligand binding, the AR undergoes a conformational change which promotes dissociation of the heat shock protein complex, dimerisation and nuclear localisation. Next, the AR binds to androgen response elements (AREs) in the regulatory regions of target genes and, via the recruitment of coactivators (proteins that enhance the receptor’s transcriptional activity) and the basal transcriptional machinery, enhances or represses gene expression [123,124,125,126].

## 6. Androgen Receptor Activity in the Lung and Regulation of TMPRSS2 and ACE2

Our understanding of AR action has predominantly come from studies that have investigated the receptor’s role in the prostate, a small secretory gland at the base of the bladder, and prostate cancer [126]. In the prostate, the AR regulates various genes important in the development and function of the gland. In prostate cancer, the AR has been shown to regulate a transcriptional profile that promotes tumour growth. However, it is well documented that the AR is expressed in, and plays an important role in, other organs [125]. For example, analysis of an AR reporter mouse demonstrated that the AR is expressed and transcriptionally active in multiple organs in male and female mice, including the testes, prostate, ovaries, uterus, salivary glands and spleen [127]. Importantly, the study also demonstrated that AR is transcriptionally active in the lung, albeit weakly. Further, microarray analysis of the AR transcriptome in the lung adenocarcinoma cell line A549 demonstrated that the AR regulates pathways involved in, e.g., oxygen transport, DNA repair and DNA recombination [128].

Several studies have investigated AR expression in the lungs, with single-cell analysis demonstrating that the receptor is expressed in multiple cell types, e.g., club cells [129,130,131]. Importantly, the AR was shown to be co-expressed with TMPRSS2 and ACE2 in AT2 cells [130], the cell type predominantly targeted by SARS-CoV-2 [132]. The AR has been shown to regulate the expression of TMPRSS2 in multiple cell types, including prostate, breast and lung cells [75,128,130,133]. Analysis of the regions upstream of TMPRSS2 identified multiple potential AREs, with a binding site approximately 13 kb upstream from the start site being crucial for optimal androgen regulation of the gene [133]. Interestingly, chromatin immunoprecipitation analysis of AR binding upstream regions of TMPRSS2 demonstrated that this regulation might be tissue-specific [130,134]. In the prostate cancer cell line LNCaP, the AR was found to bind to response elements present in approximately the first 80 kb upstream of TMPRSS2. However, in the immortalised lung cells A549 and H1944, the AR is bound to more distal regions, e.g., binding sites present approximately 100–200 kb upstream of the transcriptional start site [130]. ACE2 expression has also been shown to be regulated by the AR. For example, Baratchian et al. demonstrated that ACE2 is regulated at the RNA and protein levels by the AR in LNCaP cells [129]. Similar to TMPRSS2, AR binding sites were also identified upstream of the ACE2 gene, suggesting direct gene regulation by androgens. 

## 7. Androgen Receptor Modulators as Treatments for COVID

AR regulation of TMPRSS2 and ACE2 expression has led to speculation that androgens could contribute to SARS-CoV-2 infection and disease severity (e.g., [135]). Subsequently, it was hypothesised that inhibition of AR signalling could be a method to reduce SARS-CoV-2 infection/treat the disease [136]. Multiple inhibitors of the AR signalling pathway have been developed, and these either block androgen synthesis, also known as androgen deprivation therapies (ADT, e.g., LHRH antagonists/agonists), or bind directly to the receptor and inhibit its activity (antiandrogens). These androgen signalling inhibitors are widely used in men for the treatment of prostate cancer as well as for other diseases such as benign prostatic hyperplasia and male pattern baldness/androgenetic alopecia [137,138]. They have also been trialled/used in women to treat diseases such as breast cancer, ovarian cancer and polycystic ovarian syndrome [139,140,141,142]. 

Antiandrogens (e.g., enzalutamide) have been shown to significantly reduce ACE2 and TMRPSS2 expression in prostate and lung cell lines [130,134]. Castration of mice (removal of testicular androgen production) and treatment of mice with enzalutamide has also been shown to reduce ACE2 and/or TMPRSS2 expression in the mouse lung [130,143]. Importantly, inhibition of AR signalling has been shown to block cellular entry of pseudotyped virus expressing the SARS-CoV-2 spike protein and also the authentic virus in lung and prostate cancer cell lines [130]. However, Li et al. found that enzalutamide was unable to block SARS-CoV-2 infection in human lung organoids [134]. 

## 8. Retrospective/Observational Studies to Assess the Potential Efficacy of ADT in Relation to COVID-19

To assess the potential benefits of ADT in relation to COVID-19, several observational studies have been undertaken. The first clinical data to support the hypothesis that ADT could be useful for the management of COVID-19 came from Montopoli et al., who analysed infection rates in prostate cancer patients in the Veneto region of Italy [144]. A comparison of 5273 patients receiving ADT with 37,161 patients not receiving ADT found that although cancer patients were at higher risk of COVID-19 infection, ADT partially protected men from the disease. Soon after, a second but smaller study (22 prostate cancer patients receiving ADT and 36 that were not) performed at Mount Sinai Health System in New York City also found that ADT had potential as a treatment option for COVID-19 [145]. The study found that those receiving ADT were significantly less likely to require hospitalisation or oxygen support. However, ADT was not found to significantly reduce morbidity or intubation [145]. A more recent observational study of veterans in the US also demonstrated that men receiving ADT had reduced incidence of COVID-19 and were less likely to suffer from severe symptoms [146].

In addition to the analysis of prostate cancer patients receiving ADT, studies have also looked at the therapeutic potential of AR signalling inhibitors as a treatment option for COVID in other cohorts of men. For example, McCoy et al. investigated the potential benefit of 5-alpha-reductase inhibitors, molecules that block the conversion of testosterone into the more potent DHT, in men being treated for androgenetic alopecia such as spironolactone [147]. In agreement with the ADT studies, the authors found that there was a significant reduction in the frequency of COVID-19-related symptoms in the men receiving the 5-alpha-reductase inhibitor (n = 48) compared to those not receiving the therapy (n = 65). However, not all epidemiological studies have supported the use of ADT as a therapy for COVID-19. For example, Welén et al. investigated COVID-19 severity in prostate cancer patients in the Swedish national registers who had received different types of ADT [148]. After adjustment for age and comorbidities, the authors found no evidence that ADT protected the patients from infection, nor did it reduce the severity of the disease. 

## 9. Clinical Trials to Assess the Efficacy of ADT as a Treatment Option for COVID-19

Due to the experimental and observational clinical data investigating ADT as a therapeutic approach for COVID-19, multiple clinical trials were initiated. Using novel and experimental antiandrogen proxalutamide, one study showed significant promise in clinical trials. The trial demonstrated a significant improvement in survival and a reduction in hospitalisations in the group receiving the antiandrogen. However, concerns were raised regarding the study design, and the publication has since been retracted [138]. A second larger follow-up multicentre clinical trial was performed for proxalutamide. The trial included a total of 778 subjects who had been hospitalised with COVID-19, 423 of whom received Proxalutamide in addition to standard care. Patients who received proxalutamide were found to have a significantly better recovery rate, reduced mortality rate and, on average, spent less time in the hospital [139]. 

In contrast to the proxalutamide results, several studies investigating alternative androgen signalling inhibitors have shown limited/no benefit to patients. For example, Nickols et al. undertook a clinical trial to assess the therapeutic value of degarelix, which blocks androgen production through inhibition of the hypothalamus–pituitary signalling axis. The study was terminated after an interim analysis demonstrated that there was no significant difference in outcomes (e.g., mortality, ongoing need for hospitalisation, or requirement for mechanical ventilation) between the degarelix (plus standard care) and placebo groups [149]. Similarly, the COVIDENZA trial also found that inhibition of AR signalling had no therapeutic value for COVID-19. COVIDENZA was a randomised phase 2 clinical trial that investigated the efficacy of the antiandrogen enzalutamide. The trial enrolled 42 hospitalised COVID-19 patients, and following a safety evaluation, the trial was halted as it was found that enzalutamide-treated patients required longer stays in the hospital. A number of other clinical trials that aimed to investigate antiandrogens as a therapeutic approach for COVID-19 were subsequently withdrawn. However, at the time of writing, two proxalutamide clinical trials are ongoing (NCT05009732 and NCT04869228); hopefully, these will provide some clarity on the efficacy of targeting the AR as a treatment option for COVID-19. 

## 10. Conclusions and Further Perspectives

Considering the prohibiting cost of current COVID-19 drug regimens for low- and middle-income countries, the emerging SARS-CoV-2 variants and the COVID-19 vaccine rollout and efficacy challenges, the need for cost-effective, orally available and broad-spectrum antivirals that can act against a wide range of SARS-CoV-2 variants remains urgent [150,151]. Despite the promising antiviral effect that a range of antiandrogens display in vitro against SARS-CoV-2, the results of finalised clinical trials on the efficacy of ADT or antiandrogens in COVID-19 patients have not been conclusive enough to inform clinical practice. Various next-generation antiandrogens have been formulated, and the development of a lot more is underway, including apalutamide, darolutamide, orteronel and galeterone. These new drugs should be explored for their antiviral effects and clinical outcomes as they might be more effective against SARS-CoV-2 and perhaps more amenable for widespread use in COVID-19 patients. 

A disadvantage of antiandrogens as standalone therapeutic agents is that alternative TMPRSS2-independent virus entry pathways can counteract the inhibitory effects of antiandrogens on TMPRSS2-dependent viral entry. This might explain the mixed or inconclusive results of clinical trials to date which evaluated the monotherapy of ADT/antiandrogens in COVID-19 patients. The use of ADT/antiandrogens in combination therapy has not been evaluated so far. Combinations of antiviral drugs are more likely to function synergistically if they have distinct mechanisms of action and target different stages of the virus lifecycle. Therefore, while we wait for the outcomes of the remaining ADT clinical trials and notably proxalutamide, the next wave of investigations should be focused on the combination of antiandrogen therapy with other treatments, such as viral replication inhibitors.

In addition, it is surprising that experimental approaches to target TMPRSS2-independent virus entry pathways have been comparably limited, and the crosstalk between androgen signalling and virus pathogenicity has not been investigated thoroughly. The concept that androgens may serve dual roles in SARS-CoV-2 infection is intriguing and remains understudied. Future efforts should be focused on simultaneously targeting alternative viral entry mechanisms and defining better the mechanistic roles of androgens in the respiratory tract. In conclusion, antiandrogens have more exploratory potential as antivirals. Building on results from former clinical trials, future trials should focus on antiandrogens or ADT in combinatorial therapeutic modalities against COVID-19.
